# Empirical and Theoretical Studies on Number Comparison: Design Parameters and Research Questions

**DOI:** 10.6064/2012/858037

**Published:** 2012-06-03

**Authors:** Meltem Ballan

**Affiliations:** ^1^Center for Complex Systems and Brain Science, Florida Atlantic University, Boca Raton, FL 33431, USA; ^2^Department of Psychiatry, University of North Carolina, Chapel Hill, NC 27599-7160, USA

## Abstract

For well over one-hundred years, several key factors have been well established in the study of number comparison, including mental number line, numerical distance effect, and effect of sensory representation on number processing. The purpose of this article is to put some of these studies together to discuss design parameters and research questions addressed in the mental number comparison studies. Most of the studies discuss sensory representation and abstract number representation as well as degree of their interaction. In order to give the different views on a particular research question, the author classified studies under the related research questions. For example, Stroop and size congruity effect studies are addressed under this title chronologically. It was very clear that the design parameters and research question might change the interpretation of a task. It may be time to shift attention from the question of the interaction degree of sensory representation and abstract representation to a larger scope. The larger scope would be to understand the differences and similarities between different groups using a universal approach.

## 1. Introduction

Number comparison studies have been an interesting topic for the cognitive neuroscientists who examine the sensory representation and magnitude representation of numbers. This review article starts with the mental number line term first defined by Galton [1–3] suggesting that numbers hold a quantitative space in the mind. Almost a decade later, Moyer and Landauer [4] studied the quantitative relationships of the numbers on the mental number line. The speed of the comparison of two numbers is relative to the distance of these two numbers; the closer the numbers are the slower the responses. Moyer and Landauer [4] named this effect as distance effect. Later, Dehaene et al. [5] studied the effects of spatial representation of the numbers. Later, Dehaene and his colleagues [6, 7] named the spatiality effect of numbers as Spatial-Numerical Association of Response Code (SNARC). 

After the SNARC effect topic, three number processing models are discussed as those models have been frequently revisited to understand the effects of sensory representation on mental number comparison. The first one is the abstract-modular number processing model [8]. In this model, numbers are first converted into an abstract form, then the abstract forms are computed and transformed into a sensory model as output. The triple-code model [9] is the second model included in this review article. In this model, it is suggested that each sensory input is transformed into related abstract form in a sensory input related module (i.e., auditory numbers are transformed differently than visual numbers). Thus, the sensory input form does not affect mental number processing [9]. The third model is argues that sensory input form and mental number processing cannot be separated and those two processes interact with each other [10]. Almost all studies in number comparison agree on one of these three models.

The brief description of background studies is followed by the main topic, number comparison studies. The literature review on number comparison began with a search on PUBMED database with the key word of “number comparison” for the period of 1970 to January 2012. In order to keep the focus in adult number comparison studies, we excluded studies on disorders like acalculia, synthesia, cultural differences, and language studies along with brain imaging studies. Of the 112 articles, 24 articles that met the criteria are included in the review. 

Some of these 24 articles were putting forward the earlier findings revisiting the empirical and theoretical studies published earlier. All those that did not appear in the PUBMED database were included in the review to provide comprehensive coverage. 

With respect to theoretical and empirical results, the design parameters and the research questions are varied in the aspect of sensory representations and abstract representations of numbers. Most of the works included in this review focused on distance effect and different variations of sensory representations. The overall arguments of these studies are the degree of interaction between sensory perception, decision and motor response in the temporal and spatial domains. 

## 2. The Mental Number Line

One of the first number processing studies was conducted over 100 years ago by Galton [2, 3] in an effort to understand a common imaginary mental number line. Galton [2, 3] asked his subjects to draw lines on a paper depicting given intervals of numbers. The study concluded that subjects developed a mental representation of everyday numbers (i.e., 1,2,10, 1000) and constructed other numbers (e.g., 10,000, 250,123) on this everyday numbers domain. Galton [2, 3] suggested that a mental map of numbers automatically and involuntarily appears whenever a person makes judgments of quantity. Each mental number is assigned in an order on an imaginary line in the brain. This number line provided a sense of spatiality and quantity. 

Almost a century later, Restle [11] studied the mental number line using a systematic task. The task consisted of an addition of two numbers (A + B) and a comparison of the addition with another number (C). The numbers (A, B, and C) were ranged between 13 and 153. The results were interpreted as (i) the speed and accuracy showed an increase when A + B < 100 and C > 100 or vice versa, (ii) the speed and accuracy also increased when the difference between A and B is large and A = B, (iii) errors and latency increased by decreasing difference between A + B and C. These studies concluded that physical numbers are projected onto a mental number line and operated on that imaginary line related to their relative quantities. 

### 2.1. Distance Effect

In their seminal paper, Moyer and Landauer [4] suggested that the spatial distance between two numerals on the mental number line affects participants' reaction times (RTs). For example, comparing the numbers 9 and 5 will be faster than comparing the numbers 6 and 5. A two-sided button box was used in this experiment and participants were asked to press the key associated with larger numeral (e.g., if the larger numeral was presented on the left, the left-hand side buttons were pressed). Results showed that decision time would be approximately a linear inverse function of the numerical differences between the two numerals. In other words, the RTs decreased by the increasing distance between two numerals.

The distance effect on number comparison has been extensively studied in order to analyze comparison efficiency. In most cases, the distance effect is the dependent variable facilitated or unaffected by sensory representations. The scientists studying number comparison mainly agreed on the distance effect phenomena (e.g., [1, 4, 7, 8, 10, 12–14]). Some of these studies are discussed in the following sections.

## 3. Spatial Representation

At this point, it is clear that there is a mental number line and their relative distances from each other process numbers. But, what about spatial representation of numbers? Is there a mental spatial code for the numbers? Dehaene et al. [5] studied the spatiality of numbers. The participants were asked to judge whether the presented stimulus was numerically either larger or smaller than a reference number. The left key indicated a number larger than the reference number; the key on the right indicated a number smaller than the reference number. A second series of tasks were given, reversing the keys pressed to acknowledge a larger or smaller number compared to the reference number. An interesting pattern appeared; on average, participants respond faster when smaller numbers would indicate a left key press and also to larger numbers with a right key press. Later, Dehaene and his colleagues [6, 7] examined this effect of response side and named the finding as the Spatial-Numerical Association of Response Codes (known by its acronym, SNARC). The same effect is observed when numbers are presented either as Arabic digits (e.g., 1, 2, 5) or as verbal numbers (e.g., one, two, five), suggesting that it is determined by an abstract representation, rather than any characteristics of the visual form of the stimuli. 

## 4. Early Theories on Number Processing

McCloskey et al. [8] theoretically defined an abstract-modular number processing model. According to this model, there are three independent (sequential) modules: (i) the comprehension module is the module that physical numbers (e.g., Arabic numbers, verbal numbers) are converted into a common abstract form, (ii) calculation module is the module incorporates with number rules and facts (e.g., comparison, addition), (iii) the production module is another converter module that converts abstract forms into sensory forms (e.g., spoken numbers, written numbers) [8, 15]. 

Another alternative model discussed frequently is the triple-code model by Dehaene [9]. The model employed in this study, sensory numbers are processed in two modules, visual Arabic code and auditory Verbal code input-output modules along with a third module, the analog magnitude representation module. According to this model, numbers are converted into related abstract formats in visual Arabic code and in auditory verbal code modules and finally, the mathematical operations are performed in the analog magnitude representation module. The results are converted into a sensory output module in the same visual Arabic code and auditory verbal code modules. The model suggested that these three modules are independent and sequentially processed. Therefore, the processing, which occurs in analog magnitude representation module is not affected by a sensory model (e.g., two sensory models as 18, eighteen). 

Campbell [10] challenged abstract-modular number processing model and triple code model. This study included two experiments. In the first experiment, Campbell [10] altered the sensory input between Arabic and verbal forms in order to study the effects of written representation on the arithmetic operations (i.e., addition and multiplication). The second experiment was a number naming experiment that the input sensory numbers were in the written Arabic or verbal format and output sensory forms were auditory numbers. The results of the first experiment revealed that the response speed was faster for those numbers presented in the Arabic input model, and the accuracy in that model was higher. In addition, the speed of multiplication was slower than addition and the accuracy in multiplication was lower. The second experiment showed that the accuracy was 100% and the responses to verbal input numbers were slower than responses to Arabic input numbers. Overall, Campbell [10] concluded that the sensory input model affects the abstract representation of numerals and mathematical operations. 

## 5. Number Comparison Studies

### 5.1. Sensory Number Representation

Later, Dehaene and Akhavein [1] studied the distance effect in relation to sensory model of numerals during a numerical pair-matching task (sometimes called same-different task) in 2 conditions: quantity matching (i.e., responding yes or no to same quantity, 2-TWO or 3-TWO), or quality matching (i.e., responding yes or no to different quality, 2-TWO or 2-2). Dehaene and Akhavein [1] suggested that Arabic and verbal numerals appear to converge toward a common semantic representation of quantities. The distance effect was observed indicating that numbers were automatically converted into quantities, even when the participants had been told to attend exclusively to their physical characteristics (i.e., quality matching). As postulated by several models of number processing (e.g., [7, 13]), Arabic and verbal numerals thus appear to converge toward a common semantic representation of quantities.

Accordingly, Noel and Seron [14] discussed whether different lexico-syntactic structures are activated by exactly the same semantic representation. For example, (i) comparing the magnitude of two verbal numerals is faster when the two items share the same structure (e.g., twelve hundred, fourteen hundred) than when the two items do not share the same lexico-syntactic structure (e.g., twelve hundred, one thousand and four hundred), (ii) when calculating orally, participants tend to use the same verbal structure to express their response as the one used in the stimulus. Thus, an intermediate level of representation occurs between the input form and the semantic representation. Such an intermediate representation would express the semantic relationships that are captured by the lexico-syntactic structure through the selected lexical items and the syntactic relationships that combine them [14].

### 5.2. Stroop or Size Congruity Effect in Number Comparison

The size congruity effect (sometimes called Stroop effect) refers to the fact that physical size comparison of numerals is affected by the magnitude of the respective numeral or vice versa. Size congruity effect tasks are the most common tasks to study the automaticity in numerical processing [16]. The automaticity phenomenon suggests that human cognition is very well developed. Therefore, numbers or their physical sizes are processed without any conscious effort. 

In their study, Besner and Coltheart [17] examined the unattended physical size effect on the comparison of two numbers represented simultaneously. Besner and Coltheart [17] suggested that it is easier to make a judgment of the larger number it is physically larger than when the larger number is physically smaller. The latter was also inferred to apply to smaller numbers in relation to their congruent physical sizes. 

Henik and Tzelgov [16] modified the study by Besner and Coltheart [17] by examining two unattended variables as either physical size or quantity, in a comparative judgment task in two experiments. In the first experiment, the physical size of two one-digit number stimuli was the unattended variable incongruent or congruent to numerical distance. The unattended variable of second experiment was numerical distance as incongruent or congruent to physical size (i.e., larger physical size for smaller number quantity or smaller physical size for larger number quantity; larger physical format for larger number quantity or smaller physical format for smaller number quantity). Reaction times (RTs) were facilitated when the irrelevant dimension was congruent with the relevant dimension and were delayed when two dimensions were incongruent (size congruity effect). The response speed of physical size judgment was affected by the numerical distance between the members of the digit pair (closer the numerical distance gets, the RTs for physical judgment will be longer). This observation is interpreted to the effect that numerical distance is taken into account even when it is irrelevant to the comparative judgment being required by the task. Henik and Tzelgov [16] came to the conclusion that numerical and physical attributes of numbers are processed together. 

Fitousi and Algom [18] replicated the task developed by Henik and Tzelgov [16] to examine the size congruity effect on different distractors. In the first experiment, Fitousi and Algom [18] reproduced the same results reported by Henik and Tzelgov [16]; the distractor (i.e., physical size or numerical value) affects the decision on the number judgment or vice versa. Fitousi and Algom [18] repeated numerous experiments using a separated line beneath one or two- digit number pairs. The task was either the comparison of the numerical values or the physical size of the line. Fitousi and Algom [18] reported that size congruity effect was repeated for all experiments even with a separated line.

In the context of two-digit numbers, Foltz et al. [19] studied size congruity effect with both Arabic numbers (e.g., 32) and verbal numbers (e.g., thirty-two); for Arabic numbers the interference caused by size incongruity was greater than the facilitation caused by size congruity, whereas, for verbal numbers, the facilitation was greater than the interference. All in all, Foltz et al. [19] suggested that visual quality of the numbers affects the mental quantity judgment. 

Borgmann et al. [20] designed a proportional congruency task such that the physical size congruency level changed between 25 and 75; also, the distance changed in the range of 3 to 5. The proportion of the congruency interfered with number perception whereas it did not play any role on the physical congruity judgment. The results concluded that physical size judgment occurs before numerical judgment [20]. 

Kadosh and Henik [21] revisited the congruity effect by manipulating the luminance level of the numbers (i.e. luminance congruity). Kadosh and Henik [21] presented three types of paired numbers as congruent (the numerically larger number in darker font (e.g., 1 6)) and incongruent (numerically smaller number in the darker font (e.g., 1 6)) and neutral (both numbers represented dark (e.g., **16**)). The luminance value of the numbers was also altered to produce a logarithmic intensity function (see the model by Dehaene, [22]). The participants were instructed to pick the numerically larger number or visually darker number of two simultaneously presented numbers. The results suggested that RTs were faster: (i) when the larger number was represented in darker font than when the larger number was represented in the lighter font and (ii) when the darker number was numerically larger than when the darker number was numerically small. The conclusion was that mental number processing is perturbed by the representation of stimulus (i.e., the interaction between darkness and quantitative feature of numbers) and this process occurs automatically [21].

### 5.3. Size Congruity Effect of Dots and Cumulative Area

As it is discussed above, size congruity (or Stroop effect) has been visited frequently in Arabic and verbal number comparison studies. However, the number comparison studies on patterns, dots, and cumulative area covered by numerical values are not well studied in adults. 

Recently, Burr and Ross [23] suggested that number processing is a basic visual system feature in the context of comparing two scenes in the temporal domain. Burr and Ross [23] suggested that perceived numerosity is susceptible to adaptation, like primary visual properties of a scene, such as color, contrast, size, and speed. Number perception was decreased by adaptation to large numbers of dots on a visual display surrounding the stimulus and increased by adaptation to small numbers of dots, the effect depending entirely on the numerosity of the adaptor, not on contrast, size, orientation, or pixel density, and occurring with very low adaptor contrasts. An earlier paper by Durgin (revisited in [24]) suggested a similar conclusion, namely, that multiple information types, such as texture density and cluster, have an effect on number quantity perception. 

In the context of number comparison, Nys and Content [25] addressed the questions of whether and how numerosity affects the processing of nonsymbolic continuous dimensions (i.e., the cumulative area covered by dots). Nys and Content [25] defined the size of the dots as discrete dimension and the cumulative area covered by dots as continuous dimension. In order to address these questions, Nys and Content [25] designed a task altering the number of dots and cumulative area covered by the dots such that in one experimental condition physically small dots covered a larger cumulative area whereas, in another condition, the larger dots covered a smaller cumulative area. The relevant task question was either to make a judgment on cumulative area or size of dots, respectively. In other words, either the cumulative area judgment was cued by physical size of the dots or physical size of the dots was cued by cumulative area. The overall results supported a conclusion that task irrelevant parameter interfere with task relevant parameter. Moreover, the congruity effect showed an asymmetry, as discrete dimension was a more salient cue than continuous dimension. 

### 5.4. Priming Effect

A more recent study by Schwarz and Ischebeck [26] examined the priming effect on distance judgment. Schwarz and Ischebeck [26] hypothesized that the attributes of the prime number will interfere with the decision of the stimulus. In Schwarz and Ischebeck [26] words, the processing of stimulus presented in the nth order will be affected by the stimulus representation (in other word, prime) presented in *n* − 1 order. The task was designed to compare the numbers between 2 and 8 with a memorized reference number, 5. The parameters discussed were number repetitions (i.e., stimulus (*n*) = stimulus (*n* − 1); 2-2, 7-7) response repetition (the stimulus (*n*) was always the same; such as 3-2, 4-2, 7-2, 6-2) and response shift (stimulus (*n*) was changing; such as 3-8, 3-7, 7-4, 4-6). In the experiment 1, the Arabic number format was used to present the numbers whereas the format was altered between Arabic and verbal formats in the experiment 2. The results revealed that the processing of a stimulus is facilitated by the former stimulus (so called prime). When the former stimulus is the same as the present stimulus, the response becomes faster. This suggested that an early interaction affects the number perception stage before the distance discrimination starts [26]. 

In recent article, Naccache and Dehaene [27] challenged the direct motor specification hypothesis [28]. According to this hypothesis, each visual stimulus is predominantly associated with a particular response. In other words, the priming will affect the response of a particular stimulus when the priming is chosen only from a familiar prime set. Naccache and Dehaene [27] have chosen primes that are not associated with number task along with the primes associated with stimulus. A random letter string mask appeared followed by a prime that could be either a neutral symbol ($) or the numbers (1, 2, 3, 4, 6, 7, 8, or 9) and lastly a stimulus that could be a number among the numbers of 1, 4, 6, 9. Primes consisted of old set primes (1, 4, 6, and 9) or novel primes (2, 3, 7, and 8). The participants were instructed to compare the presented stimulus with a memorized number, 5 (smaller versus larger) and priming was presented either congruent or incongruent to the stimulus. According to the direct motor specification hypothesis, the novel primes would not interfere with the stimulus responses. However, the results showed that even if the priming is not related to the stimulus, priming either interferes with or facilitates the responses to the stimulus. Thus, Naccache and Dehaene [27] summarized that unconscious semantic priming occurs regardless of the relationship between prime and stimulus. 

Stoianov et al. [29] examined the spatial priming effect on number processing. In this study, two tasks were used; a one-digit number comparison task and a parity judgment task. The stimuli were presented sequentially between two primes presented either on the right or left of the stimulus (i.e., before-prime, 2, after-prime). In the comparison task, participants verbally answered whether the presented stimulus is larger or smaller than 5. In the parity task, the participants decided whether the presented stimulus is an odd or an even number. The results revealed that only the after-prime location interferes with number comparison and parity judgment. This suggests that visual processing of after-prime starts before response stage of processing is completed. This could very well be explained by overlapping mechanisms of visual processing and response stage of processing [29]. 

Later, a semantic alignment study was developed to examine the influence of semantic priming on number comparison [30]. The prime words were chosen either as the pair of words of few-many or as less-more. The pair of few-more is relative to the quantity whereas the pair of less-more is relative to the magnitude. The distance factor was chosen as close numbers (e.g., 2 3) and far numbers (e.g., 2 8), the stimuli were presented visually, and the prime pairs appeared before the stimulus onset and simultaneously stayed visible below the number pairs. The participants verbally responded whether or not the prime and number pairs were congruent. Magnitude primes facilitated the distance effect whereas the distance effect was not pronounced with quantity primes. Moreover, the unattended priming effect was tested when the prime and stimulus pairs were presented simultaneously, and participants were asked to ignore prime pairs and name to the larger number in the paired stimulus. The congruity effect was observed only for quantity primes (e.g., many-few, 8 2). In other words, the RTs were faster when the prime and stimulus pairs were both ascending or both descending. The authors suggested that the effect of semantic alignment on number comparison is task-dependent, and the representation of unattended semantic alignment interferes with number comparison [30]. 

### 5.5. Dual Task Effect

More recently, Sigman and Dehaene [31] discussed the different stages of number comparison. In this study, the task processing is assumed to be separated into different stages as perception, decision, and response. Sigman and Dehaene [31] hypothesized that perception and response stages can be processed simultaneously with the stages of different tasks whereas decision making cannot be shared by the stages of other tasks. The task consisted of a two-digit number comparison with a memorized reference number 45. The task complexity was controlled by two factors: format of the numbers, and number of time the experiment participants pressed the button. The number format was either Arabic (e.g., 32) or verbal (e.g., thirty-two) and the button press was either one click or two clicks. In order to examine the bottleneck stage (i.e., decision) of this task, a tone was presented and participants were also asked to pay attention this tone. The tone task consisted of two tones; high (880 Hz) and low (440 Hz). The order of tasks and shared duration were changed. For example, the tone task was presented before the number comparison task or vice versa. Overall in the number task, the RTs were facilitated by notation and distance. However, the number of times the button was pressed did not affect the RTs. The notation effect was addressed in perception stage and distance effect was addressed in decision stage, whereas the button press was addressed in the response stage. Under the assumption that only the decision making cannot be processed simultaneously, the tone task was presented: (i) at the response stage of the number task, (ii) at the stage of decision, and (iii) at the stage of perception or changing the orders of tasks. In the case of (i) the RTs of number task and tone task were very similar to the case when the number task was the only task presented. In the other cases, the RTs differed when the tasks were presented together. Thus, Sigman and Dehaene [31] suggested that numerical distance effect is processed in the decision stage, and this is the only stage that cannot be processed in parallel to any other process. Moreover, these three stages, perception, decision, and response, involved in a task are serial, and one stage cannot start until the other ends [31].

In the same context, Oriet et al. [32] suggested a similar model for dual task processing. The main task was a one-digit number comparison task; the second task was a tone task similar to Sigman and Dehaene [31] task. The keyboard keys controlled the response complexity of the number task. In half of the experiment, the participants were asked to press < for numbers smaller than 5, and > for numbers larger than 5 and this order was exchanged in the other half of experiment. The distance from the reference number 5 controlled the complexity of decision. The order of two tasks were changed in the same way explained above [31]. The results suggested that the distance effect occurs at the decision stage. The decision stage is the only stage that cannot be processed parallel to the other stages [31, 32]. 

### 5.6. Response Trajectory Effect

Santens et al. [33] challenged the suggestions that perception, decision, and response are independent serial processes. In this study, a classical one-digit number comparison task was used as the participants were asked to decide whether the presented stimulus is smaller or larger than a reference number, 5. As it was first presented by [34] a button press was presented on the active screen and participants were asked to move their index fingers to the button press either following the left trajectory or the right trajectory to the button press (e.g., left trajectory if the presented stimulus is smaller than 5). When the distance between presented stimulus and reference number is small, the moving trajectories were more curved toward the incorrect response location. The authors interpreted these trajectory differences as indicating that the response stage had started before the decision stage had been completed. 

### 5.7. Same-Different Tasks

Goldfarb et al. [35] challenged the findings suggesting that only number magnitude activates the distance effect [1, 36, 37]. In order to address this question, Goldfarb et al. [35] designed paired comparison and matching tasks. In the numerical comparison task, one digit numbers between 1 and 9 excluding 5 were chosen with the distance factors of 1,2, and 5 (e.g., 1-2, 6-8, 1-6, resp.). The paired numbers were simultaneously presented on both sides of a screen. The participants made a judgment on the presentation side (i.e., left or right side of the screen) of larger numbers. In the matching task, numerically equal number pairs were presented as well as pairs with distance factors of 1, 2, and 5. The participants were asked to judge whether the number pairs are matching (e.g., 1-1) or not (e.g., 1-2). In the numerical comparison task, classical distance effect was observed; however, the distance effect was not observed in the matching task [35]. Thus, the distance effect is task dependent, and there are cases that number magnitude does not activate distance effect (i.e., the matching task, Goldfarb et al., [35]).

Ratinckx and Fias [38] modified the same-different task (see the original task in [1]) in order to understand effects of bilateral or unilateral representations on number comparison. The number pairs had three different combinations in bilateral condition: (i) both Arabic number pairs as consistent (e.g., 1 1), (ii) both verbal number as consistent (e.g., one-one), (iii) one Arabic and one verbal numbers as inconsistent (e.g., 1-one or one-1) and in the unilateral condition the numbers were either presented on the left (e.g., 1-#, one-###) or on the right (e.g., #-1, ###-one) of the screen in relation to a fixation represented in the center. The number of # was matched to the number of letters in the verbal presentation. In order to reduce the effects of length of characters in the pairs #s were used to fill the length gaps (e.g., #1#-one, #1#-#1#) in a followup experiment. Overall results revealed that RTs for bilateral conditions are faster even if the pairs are inconsistent. The authors suggested bilateral conditions are processed as one task whereas the unilateral conditions are processed as two different tasks (i.e., dual task). The latter causes a task related competition. Moreover, these two tasks cannot be processed in parallel at the decision stage that slows the process and the variation between RTs [38].

### 5.8. Number Bisection Effect

The number bisection task is an expanded number comparison task to examine the difficulty of the task. It is basically the calculation of a numerical middle value between two presented numbers, usually called outer numbers (i.e., 5 is the numerical middle value between 3 and 7). Nuerk et al. [39] modified a task that was first used in a pilot study by Van Herten [40]. In this context, the bisection range (i.e., ranges as small, 3, 5, 7 and large, 3, 9, 15), possibility of bisection (i.e., possible 5, 7, 11 with a middle number of 8 versus impossible 5, 7, 12 with a middle value of 8.5) and numbers quantity (i.e., multipliable numbers as 2, 4, 8) were chosen as the parameters changing trial to trial. Nuerk et al. [39] aimed to examine the effects of verbal multiplication ability and the effects of outer numbers on the judgment of a middle number. The results suggested that the relationship between a middle number and outer numbers facilitates RTs; RTs were observed to be faster for the trials with the multipliable numbers than trials with nonmultipliable numbers.

### 5.9. Holistic versus Competitive Approach to Two-Digit Numbers

The studies on two-digit number comparison can be classified in three main models; (i) holistic model, in which internal representation of numbers overrides units and digits are processed as a whole (e.g., [5, 41]); (ii) sequential model, in which two-digit numbers may not be processed as a whole because both numeric components (the ones- and the tens-places) are taken into account as independent digits (e.g., [42–44]); (iii) parallel processing model, in which the ones-place digit may facilitate/interfere with tens-place digit [45–47]. 

Hinrichs et al. [43] studied two-digit numbers between 11 and 99 in two comparison tasks with a memorized reference numbers, either 50 or 55, in order to understand the ones-place effect on two-digit number comparison. In the first experiment, the memorized reference number was 55 to force participants to consider the ones-place as a part of a comparison process. The reference number in the second experiment was randomized either as 50 or as 55. The reference number 55 requires consideration of ones-place whereas reference number 50 does not require a consideration of ones-place. Results of two experiments suggested that the ones-place digit significantly affected RTs even when the tens-place digit was logically sufficient to select the correct response. The ones-place digit was not processed only in the case that memorized reference number was placed at the decade of 50. The RT results exhibited a logarithmic function of the absolute difference between the two numbers. RTs decreased as numerical distance from the reference number increased. 

In the same context, Dehaene et al. [5] investigated the numbers between 31 and 99 with a memorized reference number of 65. The participants were asked to make a judgment about the condition of the presented number as smaller or larger than 65. The results suggested that the RTs became increasingly larger as the number stimulus became closer to 65. Dehaene et al. [5] explained this logarithmical increasing RT model as an indication of the holistic process. Namely, the numbers are assigned to an abstract mental number form and this abstract form overrides the ones and tens-places along with any other sensory model. In this approach, the RTs for 31 and 35 will be exactly the same, because the second digit was not taken into account during the mental comparison.

On the other hand, Nuerk et al. [44] tested the ones-place digit and tens-place digit compatibility in a paired comparison task in which the participants were asked to compare two numbers simultaneously presented on the screen. In one scenario, the ones-place digit comparison and tens-place digit comparison did lead to the same response (e.g., both digits are larger or smaller, 52 and 67). In the second scenario, the ones-place digit comparison and tens-place digit comparison did not lead to the same response (e.g., the ones-place of the larger number is smaller, 47 and 62). The results showed that when the tens- and ones-places led to the same response (e.g., 52 and 78) the RTs were faster than was the case when the tens- and ones-places did not lead to the same response (e.g., 58 and 72). Nuerk et al. [44] suggested that the logarithmic distance effect is a very strong indicator of number perception but it does not generalize to all numbers. The study showed a competitive effect between ones-place digit and tens-place digit. Therefore, the quantity of the ones- and tens-places affected the responses as independent digits. Nuerk et al. [48] challenged their own results suggesting that the independent ones-place and tens-place theory on two-digit number comparison [44]. In this later study, Nuerk et al. [48] suggested an interaction between tens-place and ones-place using the same task parameters that were used in [44] with a slight change in the presentation of the number stimuli. In 2001, two-digit numbers were represented vertically whereas in 2004 the same numbers were presented diagonally. In this more recent study, Nuerk and his colleagues [48] agreed on the combined processing model (Stroop-like effect of ones-place on tens-place processing). Nuerk and his colleagues [48] also mentioned that the difference in the results could be related to presentation of the number stimuli. 

A year later, Zhang and Wang [47] identified three reaction time functions for three processing models, sequential, parallel and holistic. The model by Zhang and Wang [47] is based on the task developed by [43]. The reference number was 55; its tens-place was referred as *D*_*s*_ and its ones-place as *U*_*s*_. Its tens-place and ones-place digits as *D*_*t*_ and *U*_*t*_ also refer to the stimulus number, respectively. In the sequential model, the ones-places are not taken into account unless tens-places are equal. Therefore, the RTs for the numbers in the same decade will be equal in the case of *D*_*t*_ *≠D*_*s*_ (e.g., the RT for stimulus 21 and RT for stimulus 29 will be equal as only the tens- places are taken into account) and RTs will increase in the case of *D*_*t*_ = *D*_*s*_ (e.g., RT_54_ > RT_53_ as the ones- places are taken into account). The function of the sequential model looks like a step logarithmic function increasing with decreasing distance [47]. Zhang and Wang [47] suggested that two-digit number comparison is parallel and ones and tens-places interact in a way resembling the Stroop effect.

In the parallel model, the ones-place interacts with the comparison of two-digit numbers [44]. The RTs will be facilitated by ones-place digits in the case of *D*_*t*_ = *D*_*s*_ as it is in the sequential model; however, the logarithmic increase in the reaction time will be perturbed as a function of ones-place in the case of *D*_*t*_ *≠D*_*s*_. This model produces a step function perturbed by a linear function of ones-places and tens-places of stimulus and reference number [47]. 

In the holistic model, the sensory representations of the numbers do not play any role on the processing model. The mental number is abstract and perceived as a whole numerical value. The function of this model is a logarithmic model of ones-places and tens-places. In other words, the stimulus will be processed as a whole and compared with a processed whole numerical value of the reference number [5, 47]. 

Accordingly, Ganor-Stern et al. [49] and Ganor-Stern and Tzelgov [45] studied the size congruency effect of two-digit numbers using a similar compatibility approach to that used by Nuerk et al. [44]. The size congruency effect was affected by the compatibility between ones-place and tens-place digits. Thus, the physical representation of the numerals influences the quantitative judgment of the numerals. However, the effect was defined as a Stroop-like effect that ones-place digits facilitate the comparison of tens-place digits. The latter may be explained as a mixed model of abstract representation of magnitude [5] and unit-decade compatibility model [45, 47]. 

Moeller et al. [46] developed a neural-network-based model approach to identify mental two-digit number representation. Their approach included three different neural network models: (i) the numbers were represented as integrated entities; (ii) the numbers were represented as two distinct units (tens and ones places are represented as independent units); (iii) numbers were presented in the combined form, such that the distinct unit representation was in parallel to integrated representation. The results suggested that numbers are represented as the distinct unit or in the combined form. In other words, there is a competition between tens and ones places; however, the digits are not processed as individual digits; rather ones-place digits interfered with teens-place digits [45–47]. 

### 5.10. The Effects of Number Comparison on Addition

It is suggested that the numbers such as 4 + 2 and 2 + 4 are mentally represented first as the minimum and maximum addends of the addition operation and the addition is completed later [50]. Therefore, the response time for the addition of two numbers will include a function of two numbers and the comparison time of two numbers. In order to address this hypothesis, Butterworth et al [50] developed a series of tasks. The first task was a number naming task of numbers between 0 and 18. The second task was a number comparison task of the number pairs used in the third addition task. 

In general, the results revealed that number naming and number comparison are independent factors affecting number comparison (i.e., additive effect) excluding strategy used to identify 0 [50]. All in all, the suggestion is that numbers were organized in a domain-specific way and retrieved from the predominantly allocated memory units [50]. 

### 5.11. Effects of Addition on Number Comparison

In a very recent study, Charras et al. [51] examined whether summation affects the mental number comparison. Charras et al. [51] hypothesized that the sum of repeated numbers would be underestimated whereas the sum of different numbers would be overestimated. There were three types of stimulus; (i) the single numbers (e.g., 44, 64, 80), (ii) sum of repeated numbers (e.g., 22 + 22, 32 + 32, 40 + 40), and (iii) sum of different number (e.g., 24 + 20, 34 + 30, 38 + 42), along with distance factor ranging from 2 to 27. In the first experiment, the reference number was one of the numbers 48, 50, 52, and 54, which were shown in the beginning of each trial. The distance from the reference number was either 4 or 2 for the first experiment. The accuracy and the speed of the three stimulus types and two distances were analyzed. The results of first experiment suggested that RTs were slowest and accuracy was lowest for different numbers whereas the RTs were fastest and minimum wrong answers reported for single numbers. Moreover, the classical distance effect was observed. In the second experiment, the reference number, either 45 or 53, was memorized and distances from the reference number were larger in order to observe more pronounced distance effect (distance factor changed between 1 and 27). The results revealed that different number type was the most difficult condition as shown by performance (slowest RTs and more wrong answers). The results suggested that the summation of repeated numbers was estimated smaller than the summation of different numbers (e.g., 22 + 22 < 24 + 20). Overall, the speed and accuracy is overestimated for different numbers. Although this study points out a new way of examining the summation effect on number comparison, since performance with single numerals was better that with the other two conditions. Moreover, there is a suggestion that summation interfere with number comparison, as the participants performed better for single numbers was than other two conditions. The authors welcomed followup studies to discuss this matter further [51]. A further question is whether overestimation and underestimation effects are the best words to discuss this task. 

## 6. Conclusion

The studies discussed in this review and also the others on number comparison directly or indirectly address the effects of sensory representation on number processing. The model of the interaction changes from one study to another depending on the research question and study design. For example, some scientists agree that sensory representation of numbers affects numerical magnitude representation [10, 16, 21], whereas others suggest that sensory representation and magnitude representation are independent and occur sequentially [1, 13]. 

With two-digit numbers, another dichotomous processing question is asked: whether digits are processed holistically or parallel. The holistic approach suggests that mental number perception overrides units [5, 41]. Therefore, numbers are processed as a whole, whereas the combined processing approach suggests that ones-places interfere with tens-places (i.e., Stroop-like effect) [45–48].

In these contexts, there needs to be an effort to put all these findings in a common domain to develop a meta-task. A universal task model would allow scientists to understand the underlying behavior and brain mechanisms of number processing in a common domain.

Ultimately, such a meta-approach might provide some insights on mathematics or aging related disorders, for example, Dyscalculia [52, 53] and Alzheimer's diseases [54, 55].
